# Depression and post-traumatic stress disorder after aneurysmal subarachnoid haemorrhage in relation to lifetime psychiatric morbidity

**DOI:** 10.3109/02688697.2011.578769

**Published:** 2011-05-18

**Authors:** Mathilde Hedlund, Maria Zetterling, Elisabeth Ronne-Engstrom, Marianne Carlsson, Lisa Ekselius

**Affiliations:** 1 Department of Neuroscience, Psychiatry, Uppsala University Hospital, Uppsala University, Uppsala, Sweden; 2 Department of Neurobiology, Care Sciences and Society, Karolinska Institute, Stockholm, Sweden; 3 Department of Neuroscience, Neurosurgery, Uppsala University Hospital, Uppsala University, Uppsala, Sweden; 4 Department of Public Health and Caring Sciences, Uppsala University, Uppsala, Sweden

**Keywords:** Subarachnoid haemorrhage, mental disorders, depression, stress disorders, post-traumatic

## Abstract

**Introduction:**

Little is known about the roles that lifetime psychiatric disorders play in psychiatric and vocational outcomes of aneurysmal subarachnoid haemorrhage (SAH).

**Materials and methods:**

Eighty-three SAH patients without apparent cognitive dysfunction were assessed using the Structured Clinical Interview for DSM-IV axis I disorders (SCID-I) after their SAH. Diagnoses were assessed for three time periods, ‘lifetime before SAH', ‘12 months before SAH’ and ‘7 months after SAH'.

**Results:**

Forty-five percentage of patients with SAH reported at least one lifetime psychiatric disorder. After SAH, symptoms of depression and/or post-traumatic stress disorder (PTSD) were seen in 41%, more often in those with a psychiatric history prior to SAH (*p* = 0.001). In logistic regressions, depression after SAH was associated with a lifetime history of major depression, or of anxiety or substance use disorder, as well as with lifetime psychiatric comorbidity. Subsyndromal or full PTSD was predicted by a lifetime history of major depression. After the SAH, 18 patients (22%) had received psychotropic medication and/or psychological treatment, 13 of whom had a disorder. Those with a lifetime history of major depression or treatment with antidepressants before SAH had lower return to work rates than others (*p* = 0.019 and *p* = 0.031, respectively). This was also true for those with symptoms of depression and/or PTSD, or with antidepressant treatment after SAH 0 = 0.001 and *p* = 0.031, respectively).

**Conclusions:**

Depression and PTSD are present in a substantial proportion of patients 7 months after SAH. Those with a history of psychiatric morbidity, any time before the SAH, are more at risk and also constitute a risk group for difficulties in returning to work.

## Introduction

A number of studies suggest that patients afflicted with subarachnoid haemorrhage (SAH) have a considerable burden of psychopathology in its aftermath, such as depression and post-traumatic stress disorder (PTSD). The presence of depression, anxiety and PTSD is strongly related to long-term health-related quality of life.^1^,^2^ This is also true for a history of psychiatric disorders^3^ and maladaptive coping.^3^,^4^ The reported prevalence of psychopathology differs between studies. For example, the prevalence of depression varies from 5% after 2 years,^5^ 9% after 9 months^6^ and 9 years,^7^ 10% after 18 months,^8^ to 20% after 16 months.^9^ Furthermore, a systematic review of 53 observational studies indicates that about one-third of patients with stroke, irrespective of its cause, report depression.^10^ Prevalence figures for PTSD after SAH show even greater variation, with studies reporting from 6%,^6^ 19%,^11^ 32%, 12 to 60%.^8^

There are several possible explanations for these reported discrepancies. First, the patients’ level of mental well-being prior to SAH onset is regularly neglected, despite the fact that a lifetime history of psychiatric disorders is common in the general population.^13^ ^15^ Studies that overlook this fact may mistakenly blame post-SAH psychiatric problems on the SAH itself or on its consequences, when in reality, they were already present before the onset of the SAH. Furthermore, current literature suggests, from different angles, that a previous psychiatric history is an indicator of an increased vulnerability for having mental problems, particularly in connection with stressful events.^16^,^19^ Second, methodological problems such as the use of nonstandardised measures, and varying time points for assessment, also contribute to disparate findings. Third, the issue of psychological or pharmacologic treatment is not addressed as an integral factor when rates of psychiatric disorders are evaluated after SAH.

The uncertainty involved in interpreting previous reports motivates a rigorous approach to achieve a population-based SAH sample for a longitudinal analysis of psychopathology assessed by clinicianadministered semi-structured interviews using strict *Diagnostic and Statistical Manual of Mental Disorders, Fourth Edition* (DSM-IV) criteria.^20^ We therefore comprehensively assessed premorbid prevalences of lifetime and 12-month psychiatric axis I disorders in subjects consecutively admitted to a neurointensive care unit (NICU) receiving all SAH patients from a defined catchment area. In addition, we prospectively assessed the occurrence of psychiatric axis I disorders, particularly minor and major depressions as well as subsyndromal and full PTSD, on average 7 months after the SAH. We hypothesised that a history of any psychiatric disorder before the SAH predicts the occurrence of depression and PTSD 7 months after SAH.

## Materials and methods

### Participants

Consecutive patients with acute SAH, admitted to the Department of Neurosurgery, Uppsala University Hospital, between September 2002 and October 2005 were assessed. Uppsala University Hospital covers a geographical area with approximately 1,900,000 inhabitants. The incidence rates of spontaneous SAH and of ruptured aneurysms are 7.7 and 5.5/100,000/year, respectively.^21^ Inclusion criteria were the following: (i) Swedish speaking; (ii) age between 18 and 75 years; (iii) having been treated by clipping or coiling after a first time aneurysmal SAH and (iv) awake and devoid of apparent cognitive dysfunction (Reaction Level Scale (RLS85): 1-2; at the very most drowsy, but not confused).^22^ Patients admitted on a temporary basis, who had their main care elsewhere, were not included.

### Study design and procedure

The study design was prospective with a focus on those with an expected good prognosis. While still in the Department of Neurosurgery, patients were approached concerning participation in the study as soon as their medical condition allowed. Those who gave informed consent were subsequently interviewed twice. The first interview was performed within the first 10 days and concerned sociodemographic data, and the second interview was performed 7 months (mean 6.8+1.9; mean + SD) thereafter and included psychiatric status and an examination to exclude apparent cognitive dysfunction. The study complied with the principles of the Helsinki Declaration and was approved by the Uppsala University Ethics Committee.

### Outcome measures

The psychiatric outcome measure was the Structured Clinical Interview for DSM-IV axis I disorders (SCID-I).^23^ SCID-I is one of the most widely used and thoroughly researched psychiatric clinical interviews^24^ and is based on operationalised criteria defined in the DSM-IV.^25^ SCID-I interview was used to assess diagnoses for three time periods. The ‘lifetime’ prevalence is the proportion of patients in the sample who met criteria for a given diagnosis at any time in their life before and including the time of SAH onset. The ‘12-month’ prevalence is the proportion of patients in the sample who met criteria for a diagnosis at some time during the 12 months before and including the time of SAH onset. Finally, the prevalence ‘7 months after SAH’ is the proportion of patients in the sample who met diagnostic criteria for a given diagnosis at the time of the interview. This time was chosen for follow-up because it is a time when patients with an expected good outcome are likely to have ‘objectively’ recovered neurologically and to be well beyond the acute episode. Furthermore, at the chosen time point, the DSM-IV diagnostic criteria for depressive symptoms have been found to have good specificity (98%), sensitivity (100%) and a positive predictive value of 80% compared to gold standard diagnostics 6 months post-stroke.^26^ In the psychiatric interview, minor depression and subsyndromal PTSD were also registered. The DSM-IV criteria for minor depression were applied, i.e. presence of either depressed mood or loss of interest and two to four depressive symptoms and an indicator of functional impairment. A subsyndromal PTSD diagnosis was based on the criteria specified by Mylle and Maes,^27^ requiring the presence of the re-experiencing symptom cluster and at least one symptom from the avoidance and the arousal symptom clusters, and an indicator of functional impairment.^27^

All patients were interviewed by a psychiatric nurse specialist (MH), with education and training in performing SCID-I interviews and assessments. All patients were interviewed at a place of their own choice. Consequently, 53 (64%) interviews were performed at the Uppsala University Hospital, 24 (29%) in the patients’ home, 2 (2%) at another hospital, and 4 (5%) patients preferred a telephone interview.

### Statistical analysis

The clinical parameters evaluated were age, sex, the amount of blood on the first CT scan defined by the Fisher grade,^28^ the neurological condition scored using the World Federation of Neurosurgical Societies (WFNS) Committee scale^29^ and as the level on the RLS85^22^ assessed at admission to and discharge from the NICU. We also recorded whether the aneurysm was treated with surgical or endovascular technique, whether intraventricular drainage was used and whether treatment of presumed vasospasm was given.^30^ For a summary of the patients’ characteristics at baseline see Table I.

**TABLE I:** Patient characteristics

Included (*N*)	93
Death	−2
Dysphasia	−2
Did not want to participate	−2
Not available	−4

Study population	83

Males/females	30/53
Mean age in years	52 (SD = 9, range 30-74)
RLS85 at admission	
1	63
2	14
3	3
4 or 5	3
RLS85 at discharge	
1	73
2	9
3	1
Fisher grade	
I-II	22
III-IV	61
WFNS	
1	63
2	14
3	1
4 or 5	5
Ventricular drainage	30
Vasospasm	15
Type of aneurysm occlusion	
Surgery	29
Coiling	54
Work status at SAH onset	
Full-time/part time	44/22
Student	1
Retired	6
Sick leave or disability pension	10

The independent effect of psychiatric morbidity before the SAH on psychiatric morbidity 7 months after SAH was analysed using logistic regression. To increase power in the analyses, major and minor depressions on the one hand and subsyndromal and full PTSD on the other were aggregated and used as dependent variables. Clinical parameters were adjusted for in the regressions by considering them as independent covariates, provided there was a *p*-value < 0.25 in bivariate regressions.^31^ All data were analysed using SPSS 17.0.

## Results

**FIG. 1:**
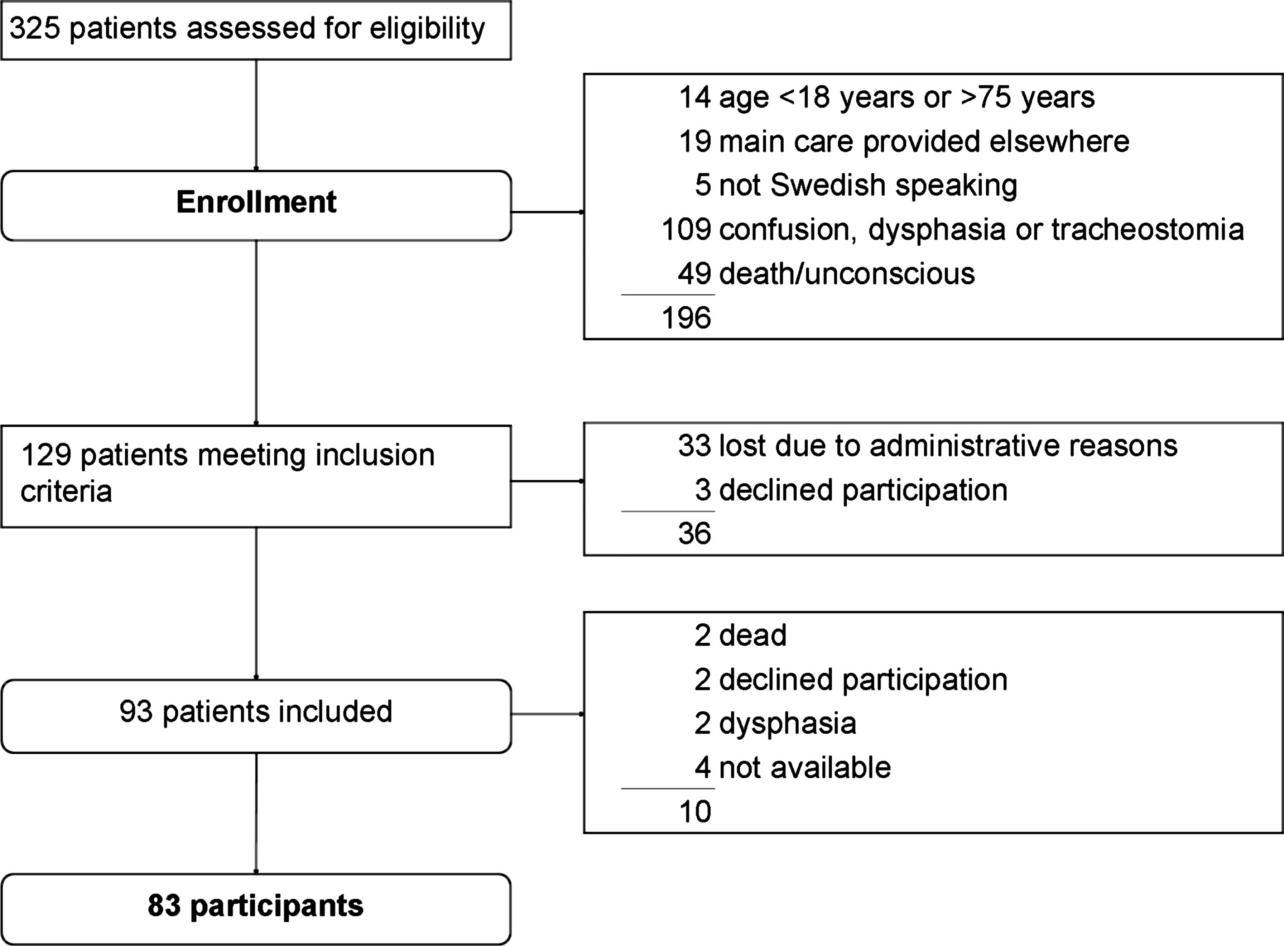
Flow chart of patients through each stage of the study presented according to the Consolidated Standards of Reporting Trials (CONSORT) statement (www.consort-statement.org/).

Of the 325 patients with SAH who were admitted during the study period, 129 met criteria for inclusion (Fig. 1). Of this group, 36 (28%) were lost before screening. Three of them declined participation, and 33 were not approached due to administrative reasons. The remaining 93 patients (72%) were included. There were no statistically significant differences between the 93 included patients and the 36 who were lost regarding sex, age or SAH characteristics (data not shown).

Ten patients were lost to follow-up when approached after 7 months. Those who were lost were older *(p = 0.02)* as compared to those not lost, but did not differ with respect to sex, WFNS or Fisher grade.

### Lifetime psychiatric disorders

Of the 83 patients, 37 (45%) presented with at least one psychiatric disorder any time before SAH; major depression and alcohol abuse or dependence were most common (Table II). Psychiatric comorbidity, defined as fulfilment of criteria for more than one disorder, was present in 17 of the 37 patients (46%).

**TABLE II:** Prevalence rates of psychiatric diagnoses in the 83 patients admitted to the NICU

	Life-time		12 months prior to SAH onset		7-month follow-up	
			
	*N*	%	*N*	%	*N*	%
Any affective disorders	22	27	4	5	17	21*
Major depressive episode	22	27	3	4	17	21
Minor depressive episode	na	na	na	na	4†	5
Manic or hypomanic episode	1	1	1	1	0	0
Dysthymia	2	2	0	0	0	0
Any anxiety disorder	22	27	11	13	26	31*
PTSD	7	8	1	1	15	18
Subsyndromal PTSD	na	na	na	na	10‡	12
Panic disorder	6	7	1	1	2	2
Social phobia	6	7	6	7	6	7
Simple phobia	6	7	6	7	6	7
Psychosis UNS (not otherwise specified)	1	1	0	0	0	0
Any substance use disorder	11	13	3	4	3	4
Alcohol abuse/dependency	10	12	2	2	2	2
Drug abuse/dependency	4	5	1	1	1	1
Eating disorder	1	1	1	1	1	1
At least one disorder	37	45	16	19	34*	41*

na, not assessed.

* Minor depression and subsyndromal PTSD not accounted for.

† Of whom, two had another disorder and one had subsyndromal PTSD.

‡ Of whom, three had another disorder and one had minor depression.

The 12-month prevalence rates for DSM-IV disorders were, as expected, lower than the lifetime prevalence rates. Sixteen (19%) of the total sample, however, fulfilled criteria for at least one diagnosis during the year preceding SAH; simple and social phobias were most common.

### Psychiatric disorders at seventh month after SAH

At seventh month after SAH, 34 patients (41%) presented with at least one psychiatric disorder, of whom, 6 were without psychiatric history before the SAH, and another eight patients met criteria for a subsyndromal diagnosis (Table II).

Major depression was the most frequent diagnosis and was present in 17 (20%) of the 83 patients. Ten of these had a lifetime history of depression, 1 of whom had an ongoing depression at the time of the SAH. Four patients experienced minor depression at the time of follow-up.

A diagnosis of SAH-related PTSD was present in 15 patients (18%) at follow-up, and another 10 patients (10%) met diagnostic criteria for subsyndromal PTSD. Of the 15 patients with full PTSD, four had experienced PTSD previously in their lifetime, one of whom had an ongoing PTSD. Seven of these 15 patients also met the DSM-IV criteria for major depression.

**FIG. 2:**
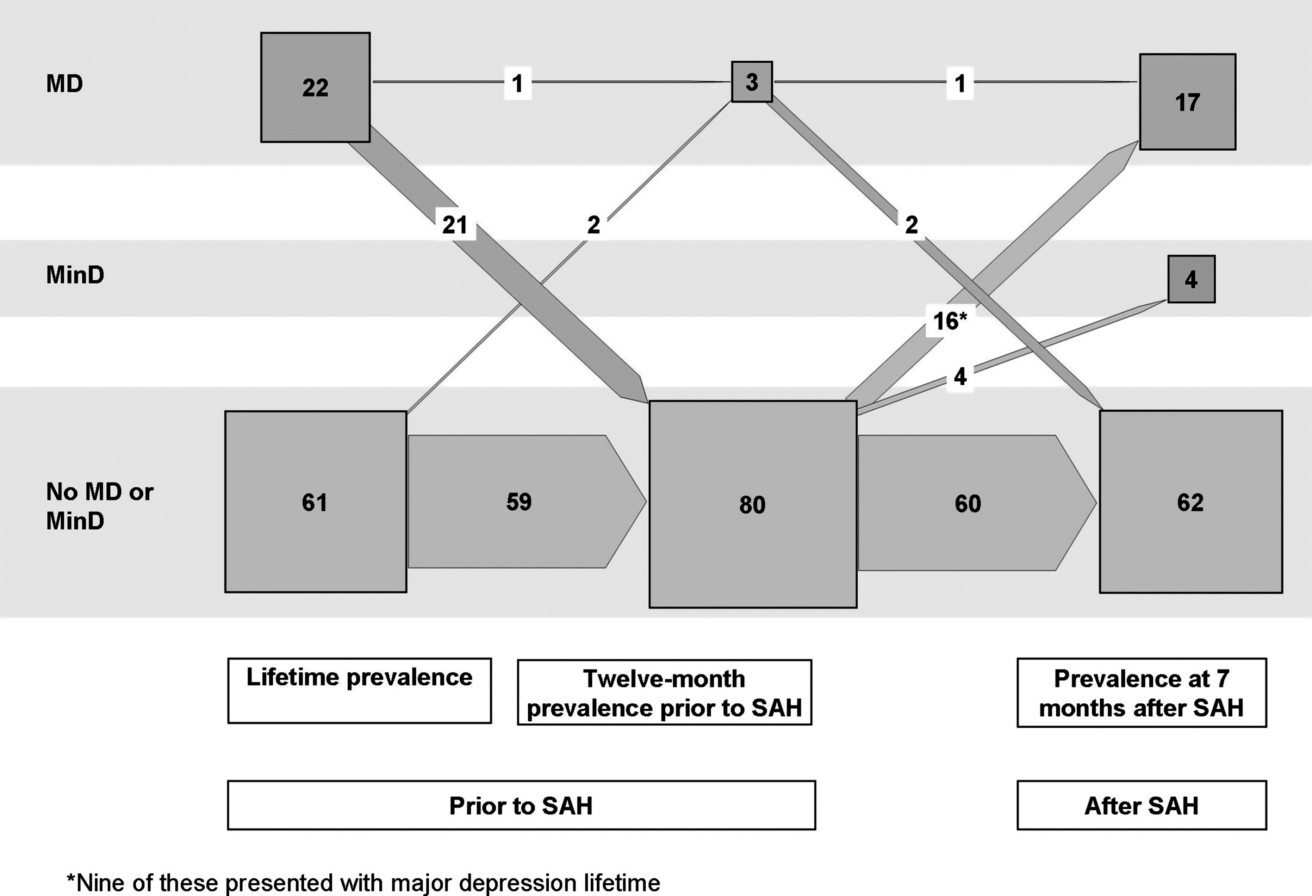
Number of patients with diagnoses of depression with respect to lifetime, 12 months prior to the SAH, and at follow-up 7 months after the SAH in the 83 investigated patients. Note: Presentation as in Schnyder *et al.^32^* Numerals are numbers of patients; sizes of squares and arrows represent quantitative proportions.

**FIG. 3:**
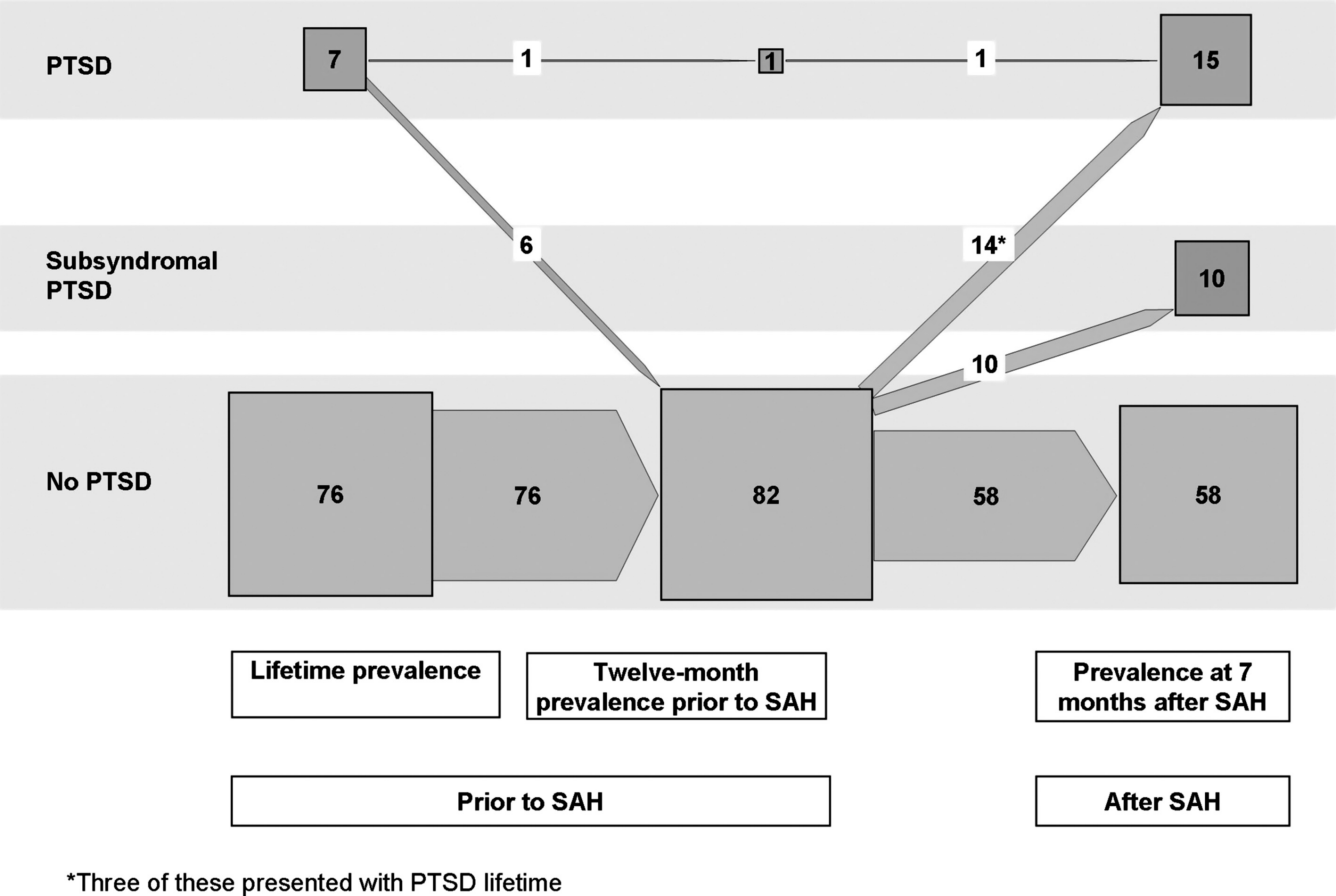
Number of patients with diagnoses of PTSD, subsyndromal PTSD and no PTSD with respect to lifetime, 12 months prior to the SAH, and at follow-up 7 months after the SAH in the 83 investigated patients. Note: Presentation as in Schnyder *et al*.^32^ Numerals are numbers of patients; sizes of squares and arrows represent quantitative proportions.

The longitudinal courses of major and minor depressions as well as PTSD and subsyndromal PTSD in the 83 patients included in the 7-month follow-up examination are portrayed in Figs. 2 and 3 using a presentation similar to that given by Schnyder U *et al*.^2^

Considered together, at follow-up, 34 (41%) patients exhibited symptoms of depression in the form of minor or major depression and/or PTSD in the form of subsyndromal or full PTSD, while 49 patients did not. Twenty-four of those 34 patients had a psychiatric history prior to the SAH compared to 13 of the 49 patients (χ^2^ = 15.8; *p* = 0.001).

Logistic regressions revealed that a depressive disorder at seventh month was predicted by the presence of a lifetime history of major depression, a lifetime anxiety disorder, a lifetime substance use disorder, any lifetime psychiatric disorder and lifetime psychiatric comorbidity (Table III). Subsyndromal or full PTSD at seventh month was only predicted by a lifetime history of major depression and of any lifetime psychiatric disorder. Similar results were obtained irrespective of whether the independent predictors (see statistics section) were forced into the models (Table III) or whether a forward stepwise regression (data not shown) was used.

**TABLE III:** Logistic regressions with the presence of minor and major depressions, as well as subsyndromal and full PTSD at follow-up 7 months after SAH as dependent variables, and the presence of lifetime psychiatric disorders before SAH as independent variables

	Minor or major depression*		Subsyndromal or full PTSD†			
		
Lifetime disorders	OR	95% CI	*p*	OR	95% CI	*p*
Lifetime affective disorder	11.9	3.0-46	0.001	7.5	2.3-24	0.001
Lifetime anxiety disorder	6.5	1.6-26	0.008	2.0	0.6-6.0	ns
Lifetime substance use disorder	9.8	1.5-66	0.019	2.0	0.5-8.0	ns
Any psychiatric disorder lifetime	14.1	3.0-66	0.001	4.4	1.5-13	0.007
Psychiatric comorbidity	10.1	2.2-47	0.003	2.1	0.6-7.1	ns

Note: Clinical predictors with *p* <0.25 in simple binary regressions were adjusted for by forcing them into regressions as independent variables. OR, odds ratio; CI, confidence interval; ns, not significant.

* Adjusted for Fisher grade, WFNS, ventricular drainage, vasospasm and female sex.

† Adjusted for WFNS and female sex.

None of the disorder groups present that 12 months prior to SAH was significantly related to a depressive disorder, or to subsyndromal or full PTSD at seventh month.

### Psychiatric treatment and work status

Nineteen patients (23%) reported a history of receiving psychiatric care before SAH onset; eight of them had been hospitalised. A total of 12 patients had previous treatment with antidepressants, six reported sedative treatment, and 1 reported treatment with antipsychotic drugs. Seven participants had ongoing antidepressant treatment at the time of SAH, five of whom did not fulfil criteria for major or minor depression at seventh month after SAH.

During the first 7 months after SAH, 18 patients (22%) had received psychotropic medication and/or psychological treatment due to psychiatric symptoms related to SAH, and 13 of them fulfilled criteria for a disorder at seventh month. Treatment was more common among those who fulfilled criteria for a lifetime psychiatric disorder (χ^2^= 17.1; *p* <0.001) or a psychiatric disorder at seventh month after SAH (χ^2^ = 9.3; *p* = 0.002).

Of the 83 patients, 67 had worked or were engaged in full-time studies prior to the SAH. Those with a lifetime history of major depression or treatment with antidepressants any time prior to the SAH had a lower return-to-work rate than the remaining patients. Thus, only two of the 25 who had a lifetime history of major depression had returned to work compared to 14 of the 42 who did not have such a history (χ^2^ = 5.5; *p* = 0.019). Seven of the 67 who had worked or were engaged in full-time studies had received antidepressant medication any time before the SAH. None of those seven were working at seventh month after SAH (χ^2^ = 4.7; *p* = 0.031).

Furthermore, those who had minor or major depression and/or PTSD in the form of subsyndromal or full PTSD at seventh month after SAH had a lower return-to-work rate than the remaining patients. Four of those 29 patients had returned to work 7 months after SAH compared to 21 of the 38 with no depressive and/or PTSD symptoms (χ^2^ = 12.1; *p* = 0.001). Finally, seven of the 67 who had worked or were engaged in full-time studies prior to the SAH had ongoing antidepressant treatment, and none had returned to work at seventh month (χ^2^ = 4.7; *p* = 0.031).

## Discussion

Key results of the present study are that psychiatric disorders are common after SAH, particularly symptoms of depression and PTSD, which affected 41 % of our patients, and that only a fraction of these patients receive treatment. Furthermore, those who exhibit a history of previous psychiatric morbidity, or treatment, constitute a group with considerable risk of inadequate adaptation after their SAH.

Forty-five percentage of the investigated patients were diagnosed with a lifetime psychiatric disorder, and 19% had suffered from at least one psychiatric disorder the year before the SAH. This is within the range of previously reported population-based epidemiological surveys from Norway^14^,^15^ and the United States.^13^

The prevalence of major and minor depressions at seventh month after SAH was 25%. This figure is in accord with what has previously been reported after stroke in general,^10^,^26^ but is higher than previously reported figures for SAH that vary from 5% after 2 years^5^ to 20% after 16 months.^9^ At least two aspects of this finding can be highlighted. First, differences in results may be the result of a ‘lack of standard methods'.^10^,^26^ Second, SAH is more common in women,^21^ who present a higher risk than men for major depression.^13^ The estimated prevalence of PTSD 7 months after SAH was 18%, which is similar to the previously reported figure of 19% after 3 months,^11^ but less than the 37% reported after 3 months and 13 months.^2^

The aggregation of minor and major depressions on the one hand and subsyndromal PTSD and full PTSD on the other was done for two reasons. First, it increased the statistical power. Second, individuals with minor depression or subthreshold PTSD exhibit clinically significant suffering. Those with minor depression often have a history of major depressive disorder and are at risk of developing major depressive disorder later on.^33^ A similar situation prevails for those with subthreshold PTSD. The PTSD concept in the DSM-IV has been criticised for being too restrictive, since a number of those afflicted who do not fulfil the diagnostic criteria for full PTSD will display clinically significant suffering^27^ and run the risk of delayed onset of full PTSD.^34^

Those with a lifetime history of psychiatric morbidity were at considerably higher risk for new symptoms after their SAH. Previous research indicates that those afflicted by psychiatric sequelae after SAH constitute a risk group for not reintegrating to their normal life.^35^ In particular, younger stroke survivors suffering from psychiatric sequelae starting within a month after their stroke seem to fail to return to work.^36^ Interestingly, in the present study, those with a lifetime history of any affective disorder prior to the SAH reported difficulties in returning to work. This indicates a possibility for identifying, as early as during acute care, a high-risk group for rehabilitation problems. Only 13 of the 34 participants in the study population who presented with a disorder at seventh month had received pharmacological or psychological treatment after SAH onset, which suggests that there is room for substantial improvement in care. Furthermore, previous research indicates that psychosocial difficulties in the aftermath of SAH are associated with gaps in community-based support.^37^ In that respect, nursing support has proven beneficial in reducing patients’ suffering.^38^ However, there is a risk that those with an expected good prognosis will miss out on nursing support.^39^,^40^ Moreover, SAH patients exposed to neurointensive care display signs of PTSD.^41^ Early psychosocial support is suggested to reduce such reactions.^41^

The study has some limitations. First, only 64% of all eligible patients could be investigated at seventh month. However, the dropouts did not differ from those included except for age. Second, the strict inclusion of participants with a good neurological status in the form of an RLS85 of 1 or 2 during acute care makes the conclusions applicable only to the group of patients with expected good outcome. Third, this study, similar to previous studies on predictors of depression in the aftermath of stroke,^42^ can be criticised for its small sample size. Fourth, studies on assessments of the occurrence of lifetime psychiatric disorders always run the risk of underreporting of prior episodes due to recall bias.^43^

The most obvious strengths of the study are its prospective design and that it is the first of its kind. The role of prior lifetime psychiatric morbidity regarding problems after SAH has not previously been assessed utilising an appropriate instrument and DSM-IV criteria.

There is need for further studies on how to organise post-SAH care in such a way that each individual is given optimal possibilities for returning to an active life in society. Underdiagnosis, and insufficient awareness of the importance of previous psychiatric history, should receive particular consideration in such studies.

## Conclusion

Depression and PTSD are present in a substantial proportion of patients 7 months after SAH. Those with a lifetime history of psychiatric morbidity before their SAH are more at risk for such conditions and also constitute a risk group for difficulties in returning to work.
